# Can Camera Traps Monitor Komodo Dragons a Large Ectothermic Predator?

**DOI:** 10.1371/journal.pone.0058800

**Published:** 2013-03-20

**Authors:** Achmad Ariefiandy, Deni Purwandana, Aganto Seno, Claudio Ciofi, Tim S. Jessop

**Affiliations:** 1 The Komodo Survival Program, Denpasar, Bali, Indonesia; 2 Komodo National Park, Labuan Bajo, Flores, Indonesia; 3 Department of Animal Biology and Genetics, University of Florence, Florence, Italy; 4 Department of Zoology, University of Melbourne, Melbourne, Victoria, Australia; Texas A&M University, United States of America

## Abstract

Camera trapping has greatly enhanced population monitoring of often cryptic and low abundance apex carnivores. Effectiveness of passive infrared camera trapping, and ultimately population monitoring, relies on temperature mediated differences between the animal and its ambient environment to ensure good camera detection. In ectothermic predators such as large varanid lizards, this criterion is presumed less certain. Here we evaluated the effectiveness of camera trapping to potentially monitor the population status of the Komodo dragon (*Varanus komodoensis*), an apex predator, using site occupancy approaches. We compared site-specific estimates of site occupancy and detection derived using camera traps and cage traps at 181 trapping locations established across six sites on four islands within Komodo National Park, Eastern Indonesia. Detection and site occupancy at each site were estimated using eight competing models that considered site-specific variation in occupancy (ψ)and varied detection probabilities (*p*) according to detection method, site and survey number using a single season site occupancy modelling approach. The most parsimonious model [ψ (site), *p* (site*survey); ω = 0.74] suggested that site occupancy estimates differed among sites. Detection probability varied as an interaction between site and survey number. Our results indicate that overall camera traps produced similar estimates of detection and site occupancy to cage traps, irrespective of being paired, or unpaired, with cage traps. Whilst one site showed some evidence detection was affected by trapping method detection was too low to produce an accurate occupancy estimate. Overall, as camera trapping is logistically more feasible it may provide, with further validation, an alternative method for evaluating long-term site occupancy patterns in Komodo dragons, and potentially other large reptiles, aiding conservation of this species.

## Introduction

Effective wildlife monitoring for conservation relies on census methods that provide good and accurate inference with low cost and high logistical efficiency [1], [2]. This is clearly challenging for apex predators which, being naturally rare and cryptic, can render many monitoring methods ineffective due to logistical, cost or behavioural issues [3]. However, given many apex predators are in decline, causing major shifts in ecosystem function, it is essential that effective population monitoring techniques be employed [4], [5], [6]. Consequently, monitoring of apex predators often relies on indirect methods (e.g. tracks and signs) to make non-validated and potentially poor inference about population status [7], [8]. For over a decade camera trapping has become a key survey method for monitoring large and often cryptic mammalian predators [9], [10], [11], [12]. When experimental design criteria and analysis assumptions are met, data collected via camera trapping can be used to estimate animal abundance via mark-recapture methods, assuming individuals can be identified. If this is not possible site occupancy can still be used to provide estimates of predator population status or distribution [13].

Whilst camera trapping is used widely for making population or community-related inference, most studies focus on mammals [13], hence the methodological practicality of using cameras to evaluate similar processes in other taxa remains relatively poorly considered. Ectothermic vertebrates such as reptiles and amphibians comprise major elements of terrestrial vertebrate communities and increasingly face similar or higher demands for robust and logistically feasible population assessment to aid their conservation; yet these groups remain largely absent from applied camera trapping studies [14].

Two reasons stand out as potentially limiting application of commonly used passive infrared camera trapping methods for making population assessment in ectothermic vertebrates. The first relates to detection capacity of the camera, which is ultimately determined by the motion/heat sensor. A passive infrared detector measures the temperature difference between an animal's body and the surrounding air which triggers photo capture. Detection performance of cameras will diminish (e.g. reduced range of detection) as ambient air temperatures increasingly match an animal's body temperature. Many terrestrial ectotherms are clearly capable of achieving body temperature sufficiently different from ambient temperature via behavioural thermoregulation [15]. Nevertheless there are still temporal and spatial processes that may prevent this from happening. For example, some ectothermic vertebrates occupy environments (e.g. dense closed forests) where it is simply too difficult to thermoregulate body temperatures above air temperature, therefore limitingcamera detection [16]. The second limiting factor is a result of the small size of many ectothermic vertebrates which makes daily body temperatures labile and again presumably reduces consistency in potential detection by camera trapping methods.

Varanid lizards are a group of reptiles which could greatly benefit from the application of camera trapping to collect data that could permit population assessment [17]. These reptiles compose a conspicuous genus of often large-bodied, predatory lizards (up to 90 kg) distributed throughout Asia, Africa and Australia [18]. Ecologically varanid lizards often function as meso- or even apex predators in vertebrate predator guilds. Multiple species face broad-scale or local population threats from direct killing for skin (used in leather products), meat and traditional medicine [19], [20], [21]. Hundreds of thousands of varanid lizards (e.g. *Varanus exanthematicus* and *V. niloticus* in Africa, and *V. salvator* in South-east Asia) are killed annually to supply the reptile leather industry [22], [23]. Exotic pet trade, habitat loss and human mediated reductions in prey have further impacted other varanid species [21], [24]. In Australia there is good evidence that invasive animals, including toxic prey and mammalian predators/competitors, are having severe to moderate impacts on different varanid species [25], [26], [27]. Further, some varanid lizards (e.g. *V. niloticus* in Florida) have become problematic invasive species with impacts via egg predation on threatened reptiles (e.g. sea turtles, terrapins, and the American crocodile) and ground-nesting birds (e.g. Florida burrowing owl) [28]. To date, documenting population trends in these lizards has often been hindered by lack of robust monitoring for similar logistical and economic reasons that hinder use of direct capture/sighting methods in carnivores.

Here we evaluate the suitability of camera trapping as a potential method for collecting presence/absence data necessary for site occupancy estimation to measure population status of a threatened large predator; the Komodo dragon (*Varanus komodoensis*). The Komodo dragon is the world's largest lizard, with adult males reaching 3 m in length and 87 kg in mass [29]. Although it is unusual for terrestrial reptiles to be apex predators, the absence of mammalian carnivores, and the enormous size of adult Komodo dragons, makes them the top predator across their range [30], [31], [32], [33]. Currently, Komodo dragons inhabit five small islands in eastern Indonesia, with four island populations located within Komodo National Park (KNP) and several fragmented populations persisting on the larger island of Flores [24]. Range size has significantly decreased in recent decades [31] with anthropogenic threats, including the poaching of Timor deer and habitat loss, suspected to be the major causes of this reduction [30], [33]. Long-term population monitoring of Komodo dragons is advocated to enable management authorities to identify those populations at risk and to instigate recovery options [30], [34].

If methodologically effective, camera trapping could greatly benefit long-term monitoring of this species. Over the last decade we have undertaken extensive cage trapping for mark-recapture studies of Komodo dragons at ten sites on four islands in Komodo National Park, and more recently the Wae Wuul Nature Reserve on Flores. For the most part, mark-recapture study via cage trapping seems effective for documenting demographic trends in this species (Komodo Survival Program, unpublished data). However, the reality of conducting ongoing long-term monitoring using current methods is finite given the heavy logistical, economic and time costs necessary to maintain such intensive monitoring.

A major perceived benefit of camera trapping methods is they may collect sufficient data to estimate annual site occupancy at existing sites and hence provide ongoing population monitoring of Komodo dragons. Indirect survey methods, such as site occupancy have been proposed as viable alternatives for assessing abundance [34], [35], [36], [37], [38] including that of large predators [39], [40], [41]. However, the first step to potentially use site occupancy estimates for inferring the population status of Komodo dragons requires testing that detection rates obtained from camera trapping are at least correlated and potentially better than detection probabilities obtained from cage trapping. Uncorrelated detection differences between camera and cage trapping methods could arise because Komodo dragons regulate their daytime active body temperature within the range 34–35.6°C for 5.1–5.6 h/day which is often within 1–2°C of ambient air temperature [42], potentially reducing the detection effectiveness of cameras.

Our study compared presence/absence data obtained using cage and camera trapping methods to estimate Komodo dragon detection probability and site occupancy. Specifically we evaluated if detection probabilities were positively correlated and ideally similar between the two methods. We then discuss the relative merits of each detection method for providing inference for population monitoring and ultimately the conservation benefits for Komodo dragons.

## Materials and Methods

### Study area

**Figure 1:**
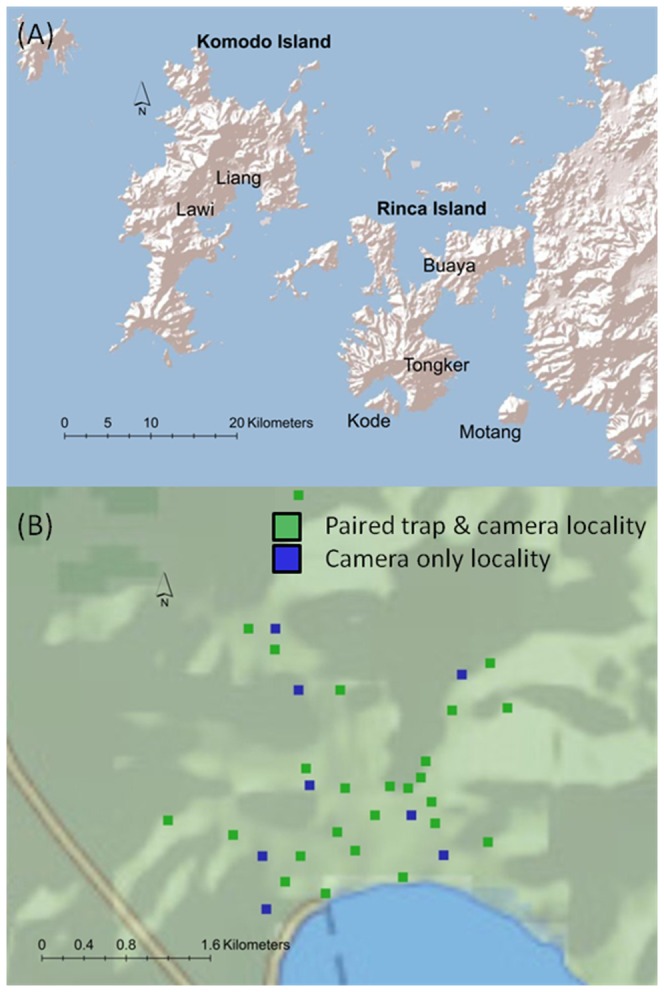
Location of study sites and trapping points. Location of six study sites on three islands within Komodo National Park were used to evaluate detection and site occupancy using cage and camera trapping for Komodo dragons. The lower panel (B) depicts a site-specific trapping design example (from the Liang site on Komodo Island) used to compare Komodo dragon detection probabilities at paired cage and camera traps or camera trap only point locations.

Fieldwork was conducted from September 2011 to March 2012 in Komodo National Park, Eastern Indonesia. Six field sites were surveyed on four islands: two each on Komodo Island (Liang, Lawi) and Rinca Island (Buaya, Tongker) and a single site on each of the small islands of Motang and Kode (Figure 1).

### Research permissions and animal ethics

This research was authorized under a collaborative research memorandum of understanding (MOU) between the Indonesian Department of Forest Protection and Nature Conservation (PHKA) and the Komodo Survival Program. Animal experimental ethics committee approval was obtained from the University of Melbourne (under Permit 0911162.1).

### Monitoring design

We used a total of 181 trapping points (i.e. a fixed location of trap placement) distributed across six study sites to conduct a Komodo dragon presence/absence survey comparing detection and site occupancy estimates using cage traps and cameras (Figure 1). These trapping points comprised long-term trap locations used annually since 2002. For 130 trapping points we used a paired method design to directly compare detection performance of cameras and traps. At an additional 51 trapping points across the six sites we used camera only locations to ensure that traps did not affect detection due to animals avoiding locations with cage traps.

For paired method comparison we utilised 8 sets of cage traps and 8 camera traps at once to monitor trapping points within each site. Within a site, cage traps and cameras were sequentially moved to new locations after three days of monitoring until all trapping points within each site were completed. Similarly, at camera only trapping point locations we utilised 3 to 4 cameras at once and again rotated them after each monitoring round to complete all camera only trapping points within a site. We conducted monitoring at each site in succession.

At each trapping point monitoring activities occurred over three consecutive days, with each trapping method checked twice daily (8–11am and 2–5pm) for the presence of Komodo dragons resulting in each locality having six sampling bouts. The time interval between the morning and afternoon daily check for each trap was ∼6 hrs. Cumulatively, the sampling design provided 1068 detection opportunities for Komodo dragons to be recorded as present or absent by each method across the six study sites.

### Detection methods

#### Cage traps

**Figure 2:**
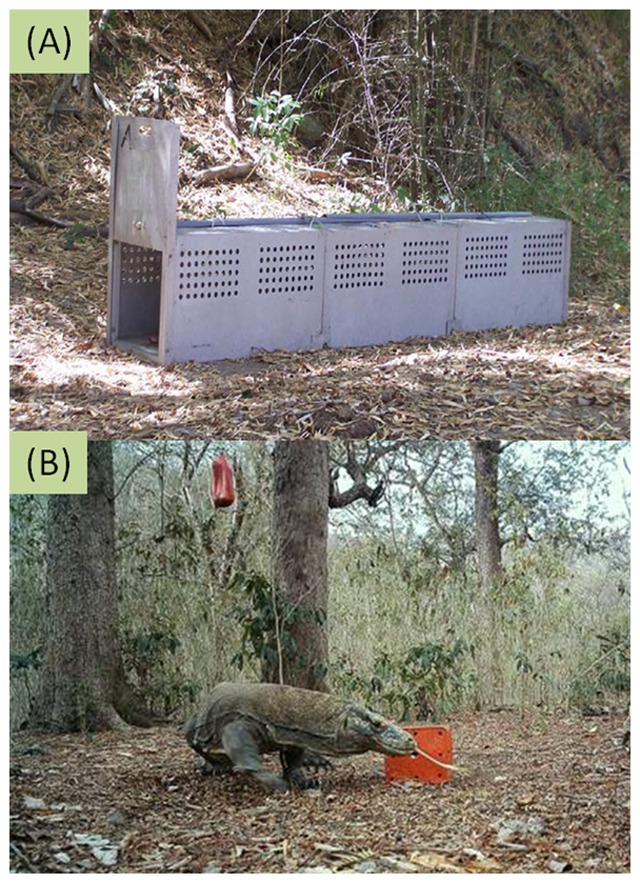
Trap setups. A photo of a cage trap (A) used to capture Komodo dragons and (B) a camera trap photo of a Komodo dragon investigating a bait box.

Within sites baited cage traps were placed at specific trapping points (Lawi, *n* = 32; Liang, *n* = 32; Buaya, *n* = 22; Tongker, *n* = 14; Motang, *n* = 16; Kode, *n* = 14) to capture Komodo dragons. Traps comprised purpose built aluminum cage traps (300 cm L×50 cm H×50 cm W; Figure 2A) fitted with a wire activated front door. The distance between trap locations was set at approximately 500 m in order to maintain independence among traps. Traps were positioned in shaded areas to avoid the potential overheating of trapped individuals. Goat meat (∼0.5 kg) was used as bait to lure lizards into traps. Additionally, a bag of goat meat was suspended 3–4 m above each trap to act as a scent lure to further attract Komodo dragons to each trapping location.

#### Camera traps

Scout Guard cameras (model SG-560V) were used in conjunction with each cage trap or in camera only trapping point locations. Where paired with cage traps, the same number of cameras (Lawi, *n* = 32; Liang, *n* = 32; Buaya, *n* = 22; Tongker, *n* = 14; Motang, *n* = 16; Kode, *n* = 14) were used as traps. The cameras were attached to a tree (40 cm above the ground) and placed 3–4 m in front of each aluminum cage trap door. The camera traps were programmed to take three photos each time the animal triggered the device. A 15 minute delay was included to prevent repeated photography of the same individual.

In addition, we used camera only trapping point locations as a control treatment to ensure there was no interaction of cage trap on camera performance (Figure 2B). Control cameras (Lawi, *n* = 8; Liang, *n* = 8; Buaya, *n* = 11; Tongker, *n* = 8; Motang, *n* = 8; Kode *n* =  8) were set and programmed as above but were also provided with a bait lure as used with cage traps. Goat meat (∼0.5 kg) was placed in aluminum boxes (25 cm L×15 cm H×15 cm W) and positioned 3–4 m in front of the camera. Similar to cage trapping additional bait (∼5 kg) was placed in a plastic bag and suspended 2–3 m above the bait box to further attract dragons to each camera only trapping point. Again, each camera was attached to a tree (40 cm above the ground) and placed within a 3–4 m radius of the baited tin, to get the best angle.

### Evaluating detection relationships between cage traps and cameras

To evaluate the relationship between the numbers of detections obtained from cage traps and cameras after each 3 day monitoring duration (comprising 6 trapping events) at the 130 paired method trapping points we considered three models comprising linear and non-linear functions.

The linear model was first considered:



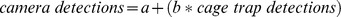



where *a* is the intercept and *b* is the slope of the relationship between camera detections and cage trap detections. As the relationship between detections obtained from the two methods could be non-linear (e.g. traps can only capture one dragon at a time compared to cameras taking photos of multiple individuals during a single sampling event), we next considered the power model:



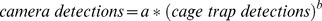



Third the logistic model was considered:



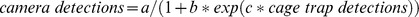



Models were fitted using WinBUGS 1.4 [41] called from the R package R2WINBUGS [43]. Parameter estimates are based on 2400 samples subsampled from 100000 samples taken from three chains after a 20000 burn-in and a thinning interval of 100, which was more than sufficient for WinBUGS to reach stationarity. Models were ranked best to worse using Deviance Information Criteria (DIC) and we also included a null (intercept only) model to further benchmark performance of the three models. As an additional diagnostic measure of model fit R^2^ was estimated for the most parsimonious model where:



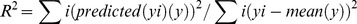



### Estimating detection and site occupancy estimates

We examined the proportion of sites occupied by Komodo dragons with a site occupancy modelling approach implemented using the program PRESENCE 4.1 [44]. This analysis is based on closed-population mark-recapture methods modified by MacKenzie *et al.* [34], [35]. The method used maximum likelihood to estimate the proportion of sites occupied by a species based on presence-absence data and adjusted for detection probability <1. Detection probabilities are denoted as *p*, while the probability that a species is present at a site (Ψ) can also be interpreted as the proportion of trapping points or sampled area occupied by Komodo dragons. The site occupancy estimates were generated using the single-season model. This model was applied because data were collected during consecutive daily sampling episodes precluding long phases of interruption in the sampling effort and hence constancy of detection is assumed [34], [35]. The major assumptions of the site occupancy single-season model are: (1) the sites are closed to change in the state of occupancy for the duration of sampling, (2) the probability of occupancy is the same for all sites, (3) species are correctly identified, (4) the probability of detecting a species at one site is independent of the probability of detecting the species at all other sites [34], [35].

To estimate detection probability and site occupancy we evaluated eight competing models including a null model (Table 1). These models considered occupancy and detection as two linked processes that could be influenced by specific variables. The main rationale for formulating these models was to evaluate factors that could conceivably influence detection probability. Thus, models (e.g. model 3; Table 1) assessed if detection probabilities differed among methods (cage traps paired with camera traps, and camera traps alone). Since meat baitsvary in condition (smell and volume) and hence allure changes over successive sampling bouts within each 3 day monitoring period, we considered models that varied detection probability across survey period (e.g. model 4; Table 1). We also considered models that varied detection probability as a function of site-specific variation to account for unspecified behavioural or environmental processes that may cause detection to vary among sites (e.g. models 6; Table 1). Additional models considered additive or interactive effects by evaluating different combinations of method, survey order and site dependent processes for influencing detection probability (e.g. models 5–8; Table 1). As yet, we are not concerned with assessing putative causes of spatial variation in occupancy (as we are currently using mark-recapture methods) so we simply constrained the occupancy term in our models to be either variable among sites (ψ (site)) or site invariant (ψ (.)). A null model (model 1, Table 1) was also included to ensure that our specified models were producing estimates better supported than by random chance alone.

**Table 1:** Summary description of models used to estimate the probability of detection (*p*) and site occupancy (ψ) of Komodo dragons at six sites in Komodo National Park, Eastern Indonesia.

Model	Model Description
1. ψ (.),*p*(.)	Occupancy and detection probability estimates are held invariant and represents the null model.
2. ψ (site),*p*(.)	Occupancy estimates vary among sites. Detection probability estimates are invariant.
3. ψ (site),*p*(method)	Occupancy estimates vary among sites. Detection probability estimate varies with method of detection (e.g. cage trap vs. camera).
4. ψ (site),*p*(survey)	Occupancy estimates vary among sites. Detection probability estimate varies as a response to survey specific attributes (e.g. time of day and bait condition).
5. ψ (site),*p*(survey+method)	Occupancy estimates vary among sites. Detection probability estimate varies as a response to survey specific attributes (e.g. time of day and bait condition) and by the method of detection (e.g. cage trap vs. camera).
6. ψ (site),*p*(site*method)	Occupancy estimates vary among sites. Detection probability estimate varies as a response to site-specific attributes (e.g. trap avoidance behaviour) interacting with method of detection (e.g. cage trap vs. camera).
7. ψ (site),*p*(site*survey)	Occupancy estimates vary among sites. Detection probability estimate varies as a response to site-specific attributes (e.g. trap avoidance behaviour) interacting with the survey-specific attributes (e.g. time of day and bait condition).
8.ψ(site),*p*(site*survey+method)	Occupancy estimates vary among sites. Detection probability estimate varies as a response to site-specific attributes (eg. trap avoidance behaviour) interacting with the survey-specific attributes (e.g. time of day and bait condition) and by the method of detection (e.g. cage trap vs. camera).

Model fit was tested on all models using a parametric bootstrap procedure to ensure that model fit was adequate and over dispersion was not present. Akaike Information Criterion (AIC) was used to rank the candidate models [45], [46]. The Akaike weight (*w_i_*) was estimated to make inference about individual model support among the candidate model set [45], [46].

## Results

### Relationship between detection probabilities from cage and camera trapping

**Figure 3:**
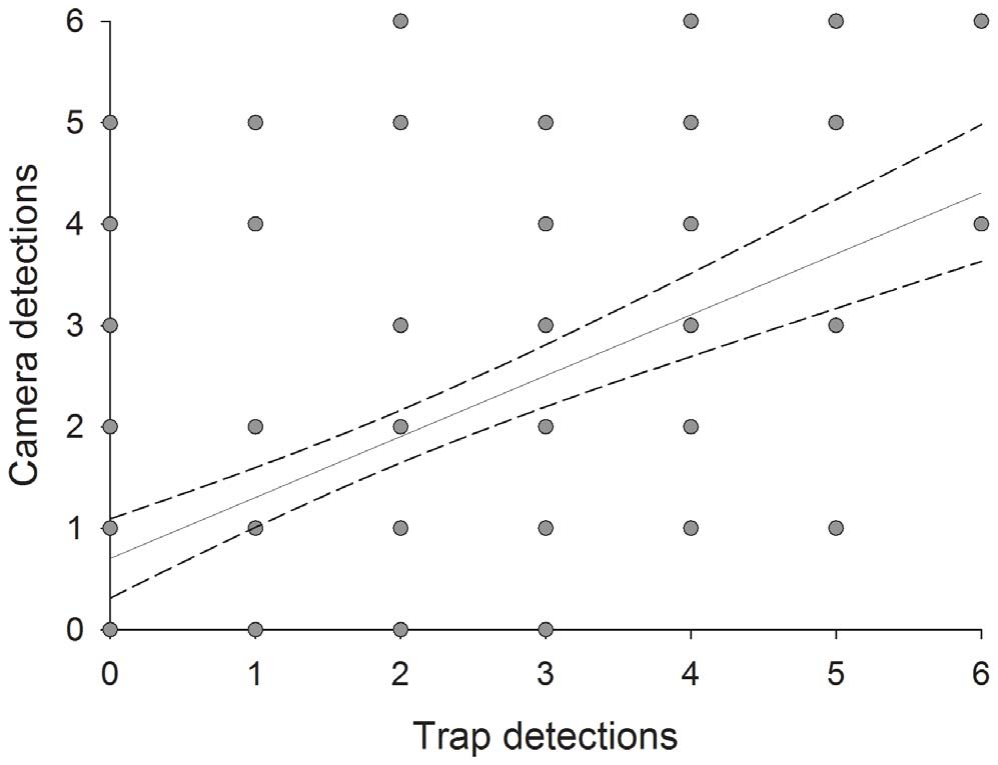
Relationship between Komodo dragon detections obtained from cage traps and cameras at all paired trapping locations across Komodo National Park. The linear regression (solid line) and associated standard errors (dashed lines) are described by the formula: *camera detection* = 0.70±0.19+0.6±0.08*cage trap detections.

A comparison of detections recorded by cage traps (mean ± SEM; 1.91±0.16 detections/trapping location; 227 total detections) and by cameras (mean ± SEM; 1.85±0.16 detections/trapping location; 228 total detections) at 130 paired sites indicated similar overall detection between methods. The relationship between detection from each method was positive (R^2^ =  0.35) and best supported by a linear regression model (camera detection  =  0.70±0.19+0.6±0.08 *cage trap detections) relative to non-linear regression and null models (Table 2; Figure 3).

**Table 2:** Ranking of linear and non-linear regression models relative to the null model examining the relationship between Komodo dragon detections obtained from cage traps and cameras.

Model	P_D_	DIC	ΔDIC	*w*
Linear	3	432.2	0.00	0.99
Power	3	452.8	20.60	0.01
Logistic	2.9	453.2	21.00	0.00
Null	2	460.9	28.70	0.00

Table describes estimated number of parameters (P_D_), Deviance Information Criterion (DIC), change in DIC (ΔDIC) relative to the most parsimonious model, and model weight (*w*).

### Model estimates of site occupancy and detection probability

**Figure 4:**
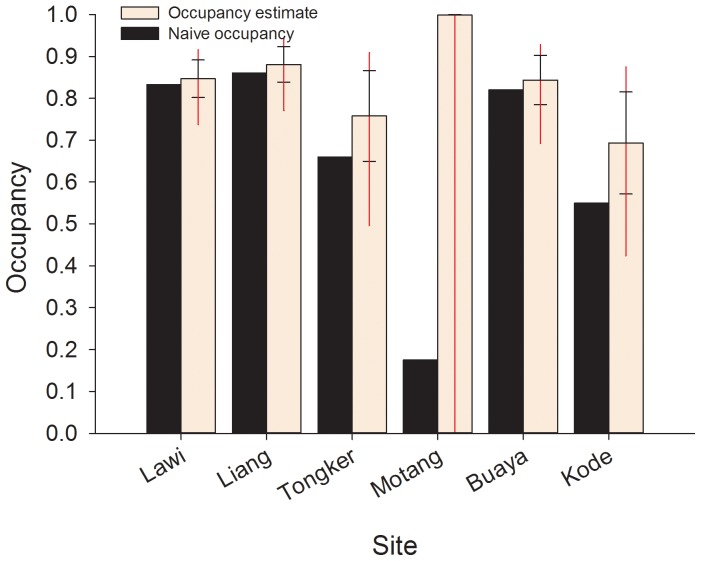
Site specific naive occupancy (black bars) and estimated site occupancy (peach bars) for Komodo dragons obtained from six sites within Komodo National Park. The capped error bars are represented by the standard error of the mean and uncapped error bars represent the upper and lower confidence limit of the mean occupancy estimated for each site. Occupancy estimates are derived from the model ψ (site),*p*(site*survey).

Naïve estimates of occupancy (i.e. % of trapping locations within each site at which lizards were detected) ranged from 0.18–0.86 (Figure 4) across the six sites.

Occupancy estimates corrected for imperfect detection were strongly supported by the model [Ψ (site), *p* (site*survey); ω = 0.74] which indicated that occupancy varied among the six sites (Table 3; Figure 4). Site occupancy estimates ranged from a low of 0.69±0.12 (95% CI = 0.42–0.87) on the small island population of Kode, to a high of 1.00 (95% CI = 0.00–1.00) on the other small island site of Motang (Figure 4). However, this high occupancy estimate was deemed extremely poor given the associated error of the 95% CI spanned 0 to 1.

**Table 3:** Summary of model-selection results based on Akaike's Information Criterion (AIC) for estimating probability of detection (*p*) and occupancy (ψ) of Komodo dragons at six site in Komodo National Park, Eastern Indonesia.

Model	AIC	ΔAIC	AIC *w*	*K*
ψ (site),*p*(site*survey)	1994.53	0.00	0.74	19
ψ (site),*p*(site*survey+method)	1996.62	2.09	0.26	22
ψ (site),*p*(site*method)	2024.41	29.88	0.00	17
ψ (site),*p*(survey)	2038.45	43.92	0.00	13
ψ (site),*p*(survey+method)	2043.82	49.29	0.00	16
ψ (site),*p*(method)	2070.56	46.15	0.00	11
ψ (.),*p*(.)	2129.26	134.73	0.00	2

Table describes Akaikie Information Criterion (AIC), change in AIC (ΔAIC) relative to the most parsimonious model, model weight (AIC *w*) and the number of parameters in each model (*K*).

**Figure 5:**
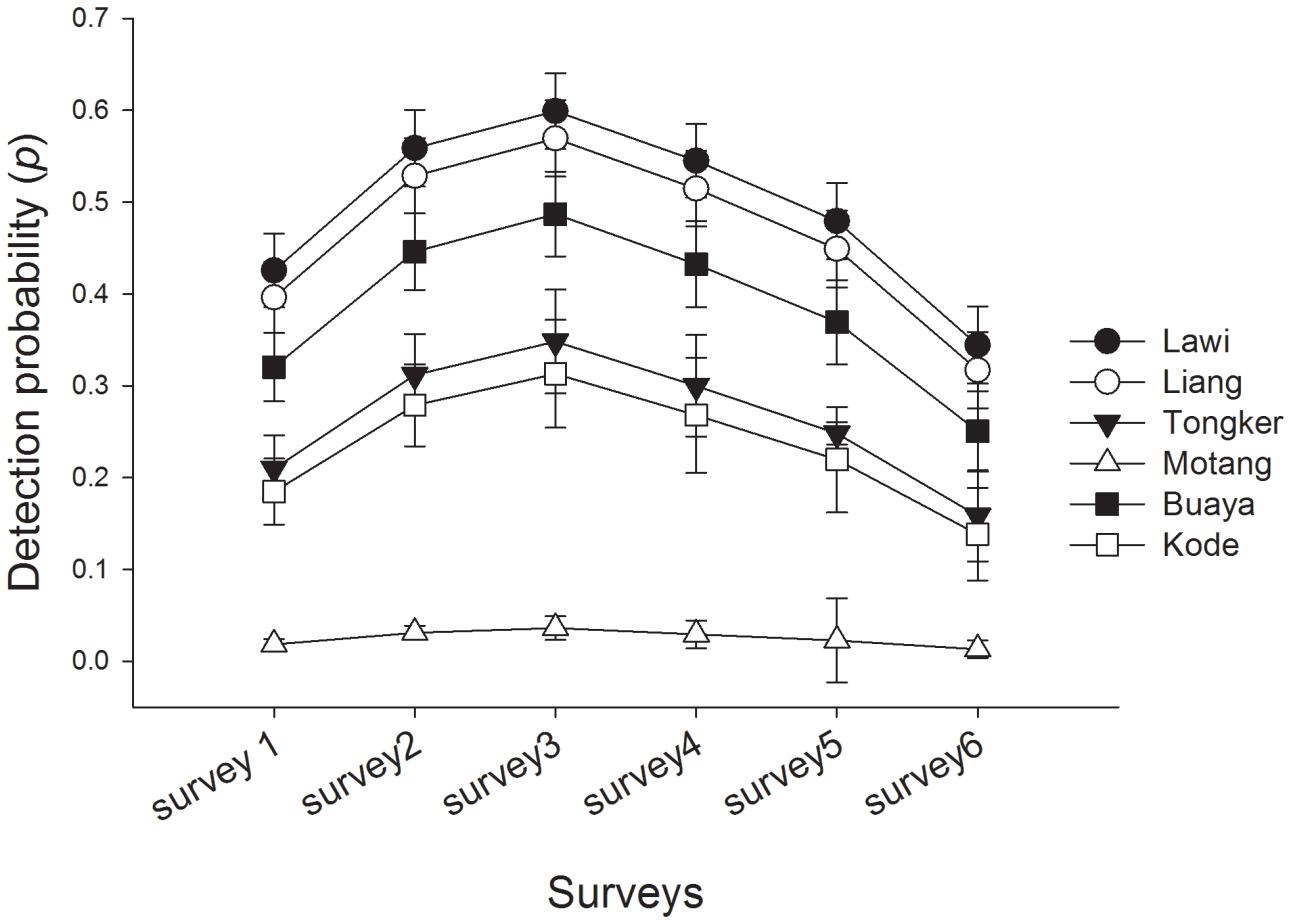
Survey-specific estimates of detection probability for Komodo dragons obtained from cumulative cage trapping and camera detections at six sites within Komodo National Park. The capped error bars represent the standard error of the mean site-specific detection estimate for each survey. The site by survey detection probability estimates are derived from the model ψ (site),*p*(site*survey).

With respect to detection, the top-ranked model indicated detection probabilities of Komodo dragons varied with site location and survey number (i.e. trapping episode). Mean detection probabilities varied among sites from a low of 0.03±0.004 on Motang, to a high of 0.49±0.04 at Lawi on Komodo Island. At all sites, except Motang, detection probabilities increased from the first trapping period, peaked at the third, then decreased until the sixth and final trapping period of each three day monitoring period (Figure 5).

The influence of trapping method was observed in the second highest ranked model, but its effect was considerably less given the ∼2 ΔAIC difference and ∼1/3 of the model weight (ω = 0.26) relative to the top model. This model's support appeared to be largely driven by the two methods producing different detection probability estimates at the Gili Motang site. Here we were unable to capture Komodo dragons using cage traps but they were detected by camera traps. This result suggested a strong behavioural aversion (i.e. trap shyness) to cage traps at this site.

## Discussion

Finding efficient and practical ways to survey apex predators is an increasing conservation imperative given widespread global population declines [5], [47]. To date there has been little consideration of the feasibility for camera traps to effectively monitor reptiles of conservation concern. This study has shown that camera trapping is an effective method for collecting presence/absence data on the vulnerable Komodo dragon.

We perceive several advantages of employing a camera-based method to monitor Komodo dragon populations over existing cage trapping. Firstly, moving to a camera-only method would considerably reduce time and labour costs and hence financial costs currently spent on trap-based Komodo dragon monitoring. Secondly, resource limitations have severely hampered managers of Komodo National Park in undertaking robust monitoring to census the status of Komodo dragon populations. Assuming provision of cameras, such a method could be employed within their existing funding to better enable them to conduct independent monitoring. The two most pressing conservation challenges facing Komodo dragons is understanding the status of populations inhabiting dwindling forests outside protected areas, and additionally the status of Komodo dragons occupying the small reserves on Flores. These reserves are being increasingly insularized by surrounding land conversion reducing habitat connectivity beyond the reserve boundary. Within these reserves, habitat quality is steadily decreasing due to increased fire frequency, a deliberate ploy used by villagers to increase pasture quality for domestic livestock. Additionally, illegal use of natural resources (timber and poaching) contributes to degrading habitat quality in reserves. Cost savings arising from replacing cage trapping with camera monitoring could permit increased monitoring in these high priority areas to better inform the population status.

Concern that eliminating direct trapping methods and ongoing mark-recapture may result in information costs by preventing estimation of key demographic parameters for Komodo dragon such as site specific survival, density, population growth and dispersal probabilities [1], [2]. Obviously mark-recapture provides data potentially enabling direct estimates of population size and vital rates necessary to make multiple inferences about the status of populations and ensuing conservation actions [1], [2]. This source of demographic information is clearly useful and arguably superior to information gathered from occupancy models. However, given increasing funding uncertainty for ongoing trapping, camera-based site occupancy methods provide the best alternative for population census of this species. Especially as increasingly sophisticated occupancy related models (e.g. multistate occupancy models) can increase the capacity for addressing more complex problems pertaining to conservation and natural resources management [48], [49]. Furthermore, there are clearly sites (e.g. Motang Island) where current cage trapping is ineffective due to trap avoidance behaviour. As cameras produce higher detection rates they may provide a viable alternative to address ongoing population monitoring at this site. Detection estimates provided by camera will still need to be increased by adding more trapping locations or increased sampling duration to improve detection sufficiently to estimate robust occupancy parameters.

Many of the logistical reasons which make camera trapping a feasible monitoring tool for large carnivores could also apply for monitoring large terrestrial reptile populations [2], [13]. However, key criteria must be met to ensure camera monitoring protocols are effective for consistent detection of reptiles. There is a general paucity of information on the population ecology of large terrestrial reptiles including lizards, tortoises and snakes. To date the IUCN has successfully evaluated only ∼39% of described reptiles, around∼21% of which are listed in IUCN categories greater than least concern [14], [50]. Standardized camera trapping methods, alongside the use of freely available software that produce site occupancy estimates (e.g. Programs: Presence [44], MARK [51] or R packages including unmarked [52], Rmark and R2Winbugs [53], [54]), offers an increasingly cost-efficient and robust analytical framework to help inventory the population status of large terrestrial reptiles.

The next major goal for us to achieve long-term Komodo dragon population monitoring is to address if site occupancy methods can provide good estimates of population status. The results presented here are considered pilot work, and irrespective of site-specific differences in occupancy estimates, they are not intended to make inference about lizard population status. We must now evaluate if our current mark-recapture study design at existing trapping locations is also adequate to provide useful site occupancy estimates obtained from camera trapping derived presence/absence data. This will mean validating different model assumptions, reducing potential detection biases present within the existing study design, and most importantly, quantifying if site occupancy estimates are sensitive and accurate enough to provide good measures of changes in population status. Efford and Dawson [55] recently demonstrated several spatial related issues that can make estimating occupancy problematic and even inadequate. In particular, poor consideration of home range characteristics of animals, or situations where the effective area of each sampling location is unknown can cause considerable bias in occupancy estimates [55]. For example, we know Komodo dragons have highly overlapping and variable home ranges. Further, as demonstrated here detection at each trapping location covaries with bait condition, leading to unknown and variable trapping areas around each detection device. These may cause biases that render occupancy estimates uninformative with respect to putative differences in population dynamics [55]. The next step in our assessment is to use trapping data collected from 234 fixed trapping locations over ten years at ten monitoring sites to estimate the relationship between both site-specific density and annual site level occupancy estimates. The nature of this relationship will enable us to determine if site occupancy is indeed an informative metric for monitoring spatial and temporal differences in Komodo dragon population status. If not, then we must first consider modifying the spatial sampling design to see if this improves the density-occupancy relationship. If this remains unsuccessful then we must trial other methods such as distance sampling to estimate differences in Komodo dragon population abundance [56].

Ultimately the choice of an appropriate metric for monitoring species of conservation concern depends on the program's objectives, scale, and resources. Here we advocate that camera-based methods, notwithstanding aforementioned issues, be considered to enable long-term Komodo dragon site occupancy estimates in lieu of otherwise costly trapping-based capture-recapture methods. We acknowledge that a transition between methods and analyses constrains access to useful demographic information to help make important inference for the conservation and ecology of Komodo dragons [29], [30], [57]. Nevertheless we perceive several clear advantages whereby camera-based monitoring could increase population monitoring via site occupancy estimates to ensure ongoing, and potentially even expanded, monitoring for Komodo dragons across their distribution. Such benefits are expected to outweigh any information loss and ultimately improve inference necessary for enhancing conservation of this iconic species.
